# Association of PD-1 gene polymorphisms and serum soluble PD-1 levels with type 1 diabetes mellitus susceptibility in a Chinese Han cohort: a case-control study

**DOI:** 10.3389/fimmu.2026.1783770

**Published:** 2026-03-27

**Authors:** Renhao Zhao, Xiuli Feng, Na Wang, Zijing Wang, Jing Liu, Guofeng Wang

**Affiliations:** 1 Department of Endocrinology, Lianyungang Clinical College of Nanjing Medical University, Lianyungang, Jiangsu, China; 2 Management of Hospital Infection, The Affiliated Lianyungang Hospital of XuZhou Medical University, The Affiliated Hospital of Kangda College of Nanjing Medical University, Lianyungang, Jiangsu, China; 3 Department of Endocrinology, The Affiliated Lianyungang Hospital of XuZhou Medical University, The Affiliated Hospital of Kangda College of Nanjing Medical University, Lianyungang, Jiangsu, China

**Keywords:** gene polymorphisms, glycated hemoglobin, PD-1/PD-L1 pathway, soluble PD-1, type 1 diabetes mellitus

## Abstract

**Background:**

The programmed cell death 1 (PD-1)/PD-L1 pathway plays a critical role in immune tolerance and has been implicated in type 1 diabetes mellitus (T1DM). However, the associations between PD-1 polymorphisms, circulating soluble PD-1 (sPD-1), and T1DM susceptibility remain unclear in Chinese populations. This study evaluated the relationships of PD-1 rs2227981 and rs2227982 variants and serum sPD-1 levels with T1DM risk.

**Methods:**

89 T1DM patients and 70 healthy controls were enrolled. Genotyping was performed using TaqMan assays. Serum sPD-1 levels were measured by enzyme linked immunosorbent assay (ELISA) in a subset (44 T1DM, 28 controls). Genetic associations were assessed using multivariable logistic regression adjusting for sex, age, uric acid (UA), and triglycerides (TG).

**Results:**

Significant differences in allele and genotype distributions of rs2227981 and rs2227982 were observed between groups. After adjustment, rs2227981 was significantly associated with T1DM under recessive (adjusted OR = 0.160, *P* = 0.027) and codominant models (adjusted OR = 6.764, *P* = 0.024). Rs2227982 remained significantly associated under additive (adjusted OR = 1.770, *P* = 0.012), dominant (adjusted OR = 0.454, *P* = 0.039), recessive (adjusted OR = 0.449, *P* = 0.032), and codominant models (adjusted OR = 0.319, *P* = 0.012). In genotype–phenotype analysis, rs2227982 was associated with HbA1c levels (*P* = 0.036). Serum sPD-1 concentrations were elevated in T1DM patients (*P* = 0.013) but showed no correlation with glycemic parameters, autoantibody status, or PD-1 genotypes.

**Conclusions:**

PD-1 rs2227981 and rs2227982 polymorphisms are associated with T1DM susceptibility in a Chinese Han cohort. Rs2227982 may be linked to glycemic phenotype, while elevated sPD-1 levels suggest altered immune checkpoint regulation rather than reflecting short-term metabolic control. Larger and longitudinal studies are needed to confirm these findings.

## 1 Introduction

Type 1 diabetes mellitus (T1DM), an autoimmune-mediated disorder characterized by progressive loss of pancreatic β-cells and consequent insulin deficiency, manifests as chronic hyperglycemia (1, 2). The global incidence of T1DM continues to rise, imposing substantial burdens on physical and psychological well-being while severely compromising patients’ quality of life (3).

Although the programmed cell death protein 1 (PD-1)/programmed death-ligand (PD-L) 1 pathway has been increasingly implicated in autoimmune diseases and immune-related diabetes, evidence directly linking PD-1 genetic variants and circulating soluble PD-1 (sPD-1) levels to T1DM susceptibility and clinical phenotypes remains limited and heterogeneous. Population-based studies simultaneously integrating PD-1 polymorphisms with soluble checkpoint phenotypes are particularly scarce, especially in East Asian cohorts (4, 5).

The costimulatory pathway mediated by PD-1 and its ligands, PD-L1 and PD-L2, delivers inhibitory signals that regulate T-cell activation, immune tolerance, and tissue damage (6, 7). PD-L1 overexpression in tumors drives immune suppression within the tumor microenvironment, enabling tumor cells to evade immune surveillance (8). Pharmacological blockade of PD-1/PD-L1 interaction improves outcomes in multiple malignancies (9). However, immune checkpoint inhibitors (ICI) therapy has been associated with immune-related adverse events, including autoimmune diabetes (10, 11), supporting a biological link between PD-1 pathway dysregulation and β-cell autoimmunity. T1DM is characterized by breakdown of peripheral tolerance, persistent activation of autoreactive T cells, and insulitis leading to progressive β-cell destruction, processes in which inhibitory immune checkpoint signaling plays a critical role in maintaining immune homeostasis (12).

PD-1 exists in membrane-bound and soluble forms (13). sPD-1 can competitively binds PD-L1 and PD-L2, thereby attenuating PD-1/PD-L–mediated inhibitory signaling (14). Elevated sPD-1 levels have been associated with disease severity in autoimmune conditions such as rheumatoid arthritis, systemic lupus erythematosus, and inflammatory bowel disease (15–17). Emerging evidence further suggests that sPD-1 may not merely reflect immune activation but can functionally modulate PD-1/PD-L1 signaling, thereby representing dynamic immune regulatory states beyond membrane-bound checkpoint signaling (18, 19). However, the role of circulating sPD-1 in T1DM remains incompletely understood, particularly in relation to genetic susceptibility and clinical phenotypes.

Single nucleotide polymorphisms (SNPs) represent the predominant form of genetic variation (20, 21). The relationship between PD-1 polymorphisms—particularly rs2227981 and rs2227982—and T1DM remains controversial (22–26). Certain PD-1 SNPs exhibit marked ethnic heterogeneity (27).

We selected rs2227981 and rs2227982 because they are common PD1 variants with biological plausibility in immune regulation, have been repeatedly investigated in autoimmune association studies, and may influence PD-1 function directly or via linkage disequilibrium.

Based on these considerations, we evaluated the association between PD-1 rs2227981 and rs2227982 polymorphisms and T1DM susceptibility and quantified serum sPD-1 levels. We hypothesized that PD-1 variants may modulate inhibitory signaling thresholds through transcriptional regulation, alternative splicing, protein structural modification, or linkage with functional loci. Concurrently, elevated sPD-1 may function as a soluble “decoy” by competing with membrane-bound PD-1 for PD-L1/PD-L2 binding, thereby potentially altering immune checkpoint signaling and influencing autoimmune responses in T1DM.

## 2 Methods

### 2.1 Subjects

This case-control study enrolled 89 patients with T1DM and 70 age- and sex-matched healthy controls (**Table 1**). Participants were consecutively enrolled between December 2020 and December 2022 at the Department of Endocrinology, Lianyungang First People’s Hospital, Jiangsu Province, China, during which fasting venous blood samples were collected from patients with T1DM. All patients met the 2023 American Diabetes Association (ADA) diagnostic criteria for T1DM. Healthy controls were selected from blood donors with normal glucose tolerance confirmed by fasting plasma glucose (<5.6 mmol/L) and glycated hemoglobin (HbA1c <5.7%) (28).

**Table 1:** Clinical and biochemical characteristics of T1DM patients and healthy controls.

Parameter	T1DM group (n=89)	Control group (n=70)	*P* value
Age (years)	43.37± 19.01	46.77 ± 12.79	0.201
HbA1c (%)	9.98 ± 2.47	5.31 ± 0.33	<0.001
FPG (mmol/L)	11.59 ± 6.46	4.88 ± 0.40	<0.001
Scr (μmol/L)	64.99 ± 45.66	65.86 ± 11.56	0.877
UA (μmol/L)	309.91 ± 120.76	313.85 ± 73.71	0.811
ALT (U/L)	17.88 ± 13.65	19.21 ± 8.08	0.469
AST (U/L)	20.36 ± 14.26	21.20 ± 5.25	0.640
TG (mmol/L)†	1.12 (0.83, 1.65)	1.08 (0.82, 1.41)	0.172
TC (mmol/L)†	4.70 (3.65, 5.35)	4.53 (4.25, 4.92)	0.430
HDL-C (mmol/L)†	1.28 (1.09, 1.48)	1.25 (1.12, 1.47)	0.881
LDL-C (mmol/L)†	2.44 (1.96, 2.88)	2.90 (2.58, 3.17)	<0.001
Sex, n (%)			0.949
male	41 (46.1)	31 (44.3)	
female	48 (53.9)	39 (55.7)	

T1DM, type 1 diabetes mellitus; HbA1c, glycated hemoglobin; FPG, fasting plasma glucose; Scr, serum creatinine; UA, uric acid; ALT, alanine aminotransferase; AST, aspartate transferase; TG, triglycerides; TC, total cholesterol; HDL-C, high-density lipoprotein cholesterol; LDL-C, low-density lipoprotein cholesterol. Data are presented as mean ± standard deviation (SD) for normally distributed variables, and between-group comparisons were performed using the independent-samples t test. Non-normally distributed variables (marked with †) are presented as median (interquartile range, IQR), and were compared using the Mann–Whitney U test. Categorical variables are expressed as number (percentage) and were compared using the χ^2^ test or Fisher’s exact test where appropriate.

The study protocol was approved by the Ethics Committee of Lianyungang First People’s Hospital (Approval number: KY-20221115001-02) and registered with the Chinese Clinical Trial Registry (ChiCTR2300079096). Written informed consent was obtained from all participants in accordance with the Declaration of Helsinki.

### 2.2 Autoantibody testing

Insulin autoantibody (IAA), glutamic acid decarboxylase antibody (GADA) and islet antigen-2 autoantibody (IA-2A) were measured using chemiluminescent immunoassays in the hospital clinical laboratory, and results were retrieved from the hospital laboratory information system. IA-2A was assessed as part of routine clinical evaluation; however, due to the limited number of IA-2A–positive cases in this cohort, IA-2A was not analyzed separately in the statistical comparisons. For genotype–phenotype analyses, overall autoantibody positivity, defined as positivity for any of the tested islet autoantibodies, was considered.

### 2.3 SNPs selection and genotyping

Two SNPs in PD-1 were selected from the National Center for Biotechnology Information (NCBI) database. All of the SNPs satisfied the following criteria: (1) SNPs that are associated with autoimmune disease occurrence or development according to the results of existing research; (2) SNPs that may influence function or expression of PD-1 and PD-L1 by NCBI; (3) minor allele frequency (MAF) ≥ 5% in Chinese Han population (22).

Peripheral blood samples (5 mL) were collected in Ethylene Diamine Tetraacetic Acid (EDTA) anticoagulant tubes and centrifuged within 2 hours post-collection. Plasma and buffy coat fractions were aliquoted and stored at -80 °C until analysis. Genomic DNA was isolated using the Solarbio Blood Genome DNA Extraction Kit (Solarbio Science & Technology Co., Beijing, China). DNA purity (OD 260/280) ranged between 1.6 and 1.8, and DNA concentration exceeded 0.2 ng/μL. Samples not meeting predefined quality criteria were excluded prior to genotyping.

PD-1 rs2227981 and rs2227982 genotyping was performed via TaqMan probe-based real-time PCR (Thermo Fisher Scientific, Waltham, MA, USA) on an Applied Biosystems 7500 Real-Time PCR System. Probe sequences were as follows:

rs2227981: 5′-GTGGCTGGGCACTCCGAGGGCCGTC[A/G]GCTGAGCCCCTGCGGGCGGGGGATG-3′ (VIC/FAM).
rs2227982: 5′-ATAGTCCACAGAGAACACAGGCACG[G/A]CTGAGGGGTCCTCCTTCTTTGAGG-3′ (VIC/FAM).

Genotyping quality control procedures were implemented as follows: (1) both the overall sample call rate and SNP call rates exceeded 99%; (2) Hardy–Weinberg equilibrium (HWE) was assessed in the control group using the χ^2^ test to evaluate genotyping quality; (3) approximately 5% of samples were randomly selected for duplicate genotyping, and the concordance rate was 100%; (4) laboratory personnel performing genotyping were blinded to case/control status.

### 2.4 Measurement of serum sPD-1

Serum sPD-1 concentrations were quantified in a subset of participants (44 T1DM patients and 28 controls) using a commercial enzyme-linked immunosorbent assay (ELISA) kit (EH0252; Wuhan Fine Biotech Co., Wuhan, China) following manufacturer protocols.

### 2.5 Statistical analysis

All statistical analyses were performed using IBM SPSS Statistics 26.0 (Armonk, NY, USA) and GraphPad Prism 9.5.1 (San Diego, CA, USA). Continuous variables were expressed as mean ± standard deviation (SD) for normally distributed data or median (interquartile range, IQR) for non-normally distributed data, while categorical variables were reported as frequency (%). Normality of continuous variables was assessed prior to analysis. Between-group comparisons utilized the following tests: independent two-sample Student’s t-test for normally distributed variables, Mann-Whitney U test for non-normally distributed variables, and χ^2^ test or Fisher’s exact test for categorical data where appropriate. For comparisons of continuous clinical variables among genotype groups (AA, AG, and GG), one-way analysis of variance (ANOVA) was used for normally distributed variables, whereas the Kruskal–Wallis H test was applied for non-normally distributed variables. When the overall ANOVA was statistically significant (*P* < 0.05), Bonferroni-adjusted pairwise comparisons were conducted to control the family-wise error rate. HWE was assessed for all SNPs in the control group via χ^2^ tests. Genetic associations with T1DM susceptibility were evaluated through multivariable binary logistic regression under additive, dominant, and recessive, and codominant models, with T1DM status (1 = T1DM, 0 = control) as the dependent variable, adjusting for sex, age, uric acid (UA), and triglycerides (TG), with results presented as adjusted odds ratios (ORs) and 95% confidence intervals (CIs). Correlations between serum sPD-1 levels and clinical parameters were analyzed using Pearson’s correlation for normally distributed continuous variables and Spearman’s rank correlation for non-normally distributed or ordinal variables, with correlation coefficients reported as r. A two-tailed *P*-value <0.05 defined statistical significance.

## 3 Results

### 3.1 Clinical and biochemical characteristics of study participants

Demographic and biochemical profiles of the 89 T1DM patients and 70 healthy controls are summarized in **Table 1**. Compared with healthy controls, patients with T1DM exhibited significantly elevated levels of HbA1c (*P* < 0.001) and fasting plasma glucose (FPG) (*P* < 0.001), alongside significantly reduced low-density lipoprotein cholesterol (LDL-C; *P* < 0.001). No significant intergroup differences were observed in sex, age, serum creatinine (Scr), UA, alanine aminotransferase (ALT), aspartate aminotransferase (AST),TG, total cholesterol (TC), high-density lipoprotein cholesterol (HDL-C) (all *P >*0.05).

### 3.2 HWE testing for PD-1 rs2227981 and rs2227982 in healthy controls

HWE was assessed in the control group to evaluate genotyping quality. Both rs2227981 (*P* = 0.166) and rs2227982 (P = 0.296) conformed to HWE (both *P* > 0.05) (**Table 2**).

**Table 2:** HWE testing for PD-1 rs2227981 and rs2227982 in healthy controls.

Gene	SNP	Chromosome	Position	Region	Alleles	MAF	HWE *P*-value
PD-1	rs2227981	2	241851121	exon 5	G/A	A=0.24	0.166
PD-1	rs2227982	2	241851281	exon 5	G/A	G=0.43	0.296

HWE, Hardy-Weinberg equilibrium; PD-1, programmed cell death protein 1; SNP, single nucleotide polymorphism; MAF, minor allele frequency. Notes: HWE was assessed in healthy controls using the χ^2^ test.

### 3.3 Allelic and genotypic associations of PD-1 rs2227981 and rs2227982 with T1DM

Significant differences in both allelic and genotypic frequencies were observed between T1DM patients and healthy controls at the PD-1 rs2227981 and rs2227982 loci. For rs2227981, the A allele frequency was significantly higher in T1DM patients than in controls (36.0% vs. 24.3%; χ^2^ = 5.005, *P* = 0.025), suggesting that the A allele was associated with increased susceptibility to T1DM. Similarly, for rs2227982, the G allele was more frequent in T1DM patients compared with controls (59.6% vs. 42.9%; χ^2^ = 8.752, *P* = 0.003), indicating an association between the G allele and increased T1DM susceptibility. Genotype distributions for both SNPs also differed significantly between groups (rs2227981: χ^2^ = 6.406, *P* = 0.041; rs2227982: χ^2^ = 7.671, *P* = 0.022) (**Table 3**). These associations were further evaluated using multivariable logistic regression models (**Table 4**).

**Table 3:** Allelic and genotypic frequencies of PD-1 rs2227981 and rs2227982 in T1DM patients and healthy controls.

SNP	Group	Allele frequency (%)		Genotype frequency (%)		
		A	G	AA	AG	GG
rs2227981	T1DM (n=89)	64(36.0)	114(64.0)	12(13.5)	40(44.9)	37(41.6)
	Controls (n=70)	34(24.3)	106(75.7)	2(2.9)	30(42.9)	38(54.3)
	*P* value	χ^2^ = 5.005, *P* = 0.025		χ^2^ = 6.406, *P* = 0.041		
rs2227982	T1DM (n=89)	72(40.4)	106(59.6)	17(19.1)	38(42.7)	34(38.2)
	Controls (n=70)	80(57.1)	60(42.9)	25(35.7)	30(42.9)	15(21.4)
	*P* value	χ^2^ = 8.752, *P* = 0.003		χ^2^ = 7.671, *P* = 0.022		

PD-1, programmed cell death protein 1; T1DM, type 1 diabetes mellitus; SNP, single nucleotide polymorphism. Comparisons of allele and genotype frequencies between groups were performed using the χ^2^ test.

**Table 4:** Association of PD-1 rs2227981 and rs2227982 with T1DM susceptibility under different genetic models.

SNP	Model	OR (95% CI)	*P*	OR (95% CI)*	P*
rs2227981	Additive	1.800(1.078-3.007)	0.025	1.692(0.991-2.889)	0.054
	Recessive	0.189(0.041-0.873)	0.033	0.160(0.032-0.811)	0.027
	Dominant	0.599(0.319-1.127)	0.112	0.683(0.353-1.320)	0.257
	Codominant	6.162(1.290-29.439)	0.023	6.764(1.284-35.631)	0.024
rs2227982	Additive	1.826(1.184-2.814)	0.006	1.770(1.133-2.766)	0.012
	Recessive	0.441(0.216-0.900)	0.025	0.449(0.216-0.933)	0.032
	Dominant	0.425(0.207-0.873)	0.020	0.454(0.214-0.961)	0.039
	Codominant	0.300(0.126-0.713)	0.006	0.319(0.131-0.780)	0.012

PD-1, programmed cell death protein 1; T1DM, type 1 diabetes mellitus; SNP, single nucleotide polymorphism; OR, odds ratio; CI, confidence interval. Notes: Binary logistic regression was performed with T1DM as the dependent variable (case = 1, control = 0). OR*、P* were adjusted for sex, age, uric acid (UA), and triglycerides (TG). Genetic models were coded as follows: rs2227981: Risk allele = A, reference genotype = GG. Additive model: GG = 0, AG = 1, AA = 2; Dominant model: AA+AG=1, GG = 0; Recessive model: AA = 1, AG+GG=0; Codominant model with GG as reference; rs2227982: Risk allele = G, reference genotype = AA. Additive model: AA = 0, AG = 1, GG = 2; Dominant model: GG+AG=1, AA = 0; Recessive model: GG = 1, AA+AG=0; Codominant model with AA as reference. Additive models assume a per-allele effect. Dominant models compare heterozygous plus minor homozygous vs major homozygous. Recessive models compare minor homozygous vs others. Codominant models treat genotypes as categorical variables.

### 3.4 Genetic model analysis of PD-1 rs2227981 and rs2227982 in T1DM susceptibility

Multivariable logistic regression was performed under additive, recessive, dominant, and codominant models to assess the association of PD-1 polymorphisms with T1DM susceptibility. For rs2227981 (risk allele = A, reference genotype = GG), significant associations were observed in the additive (OR = 1.800, 95% CI: 1.078–3.007, *P* = 0.025), recessive (OR = 0.189, 95% CI: 0.041–0.873, *P* = 0.033), and codominant (OR = 6.162, 95% CI: 1.290–29.439, *P* = 0.023) models. After adjusting for sex, age, UA, and TG, the recessive (OR* = 0.160, 95% CI*: 0.032–0.811, P* = 0.027) and codominant (OR* = 6.764, 95% CI*: 1.284–35.631, P* = 0.024) models remained significant, while the additive model approached significance (OR* = 1.692, 95% CI*: 0.991–2.889, P* = 0.054).

For rs2227982 (risk allele = G, reference genotype = AA), all models demonstrated significant associations: additive (OR = 1.826, 95% CI: 1.184–2.814, *P* = 0.006), dominant (OR = 0.425, 95% CI: 0.207–0.873, *P* = 0.020), recessive (OR = 0.441, 95% CI: 0.216–0.900, *P* = 0.025), and codominant (OR = 0.300, 95% CI: 0.126–0.713, *P* = 0.006). After adjustment, all models retained significance: additive (OR* = 1.770, 95% CI*: 1.133–2.766, P* = 0.012), dominant (OR* = 0.454, 95% CI*: 0.214–0.961, P* = 0.039), recessive (OR* = 0.449, 95% CI*: 0.216–0.933, P* = 0.032), and codominant (OR* = 0.319, 95% CI*: 0.131–0.780, P* = 0.012) (**Table 4**).

### 3.5 Association of rs2227981 and rs2227982 genotypes with clinical characteristics in T1DM patients

Analysis of clinical characteristics in T1DM patients stratified by rs2227981 genotypes revealed no significant associations with HbA1c, FPG, or autoantibody positivity rates across AA (n=12), AG (n=40), and GG (n=37) genotypes (all *P* > 0.05; **Table 5**).

**Table 5:** Clinical characteristics of T1DM patients with different genotypes at rs2227981.

Parameter	AA(n=12)	AG(n=40)	GG(n=37)	F	*P* value
HbA1c (%)	10.53 ± 3.05	10.33 ± 2.45	9.41 ± 2.25	1.713	0.186
FPG (mmol/L)	12.8 ± 6.07	12.36 ± 6.83	10.37 ± 6.12	1.168	0.316
Autoantibody positivity (n)	7(58.3)	32(80.0)	27(73.0)		0.333

T1DM, type 1 diabetes mellitus; HbA1c, glycated hemoglobin; FPG, fasting plasma glucose. Notes: Continuous variables are presented as mean ± standard deviation (SD) and were compared using one-way analysis of variance (ANOVA). Categorical variables are presented as number (percentage) and were compared using the χ^2^ test or Fisher’s exact test where appropriate. Autoantibody positivity was defined as positivity for at least one of insulin autoantibody (IAA), islet antigen 2 autoantibody (IA-2A), and glutamic acid decarboxylase autoantibody (GADA).

**Figure 1:**
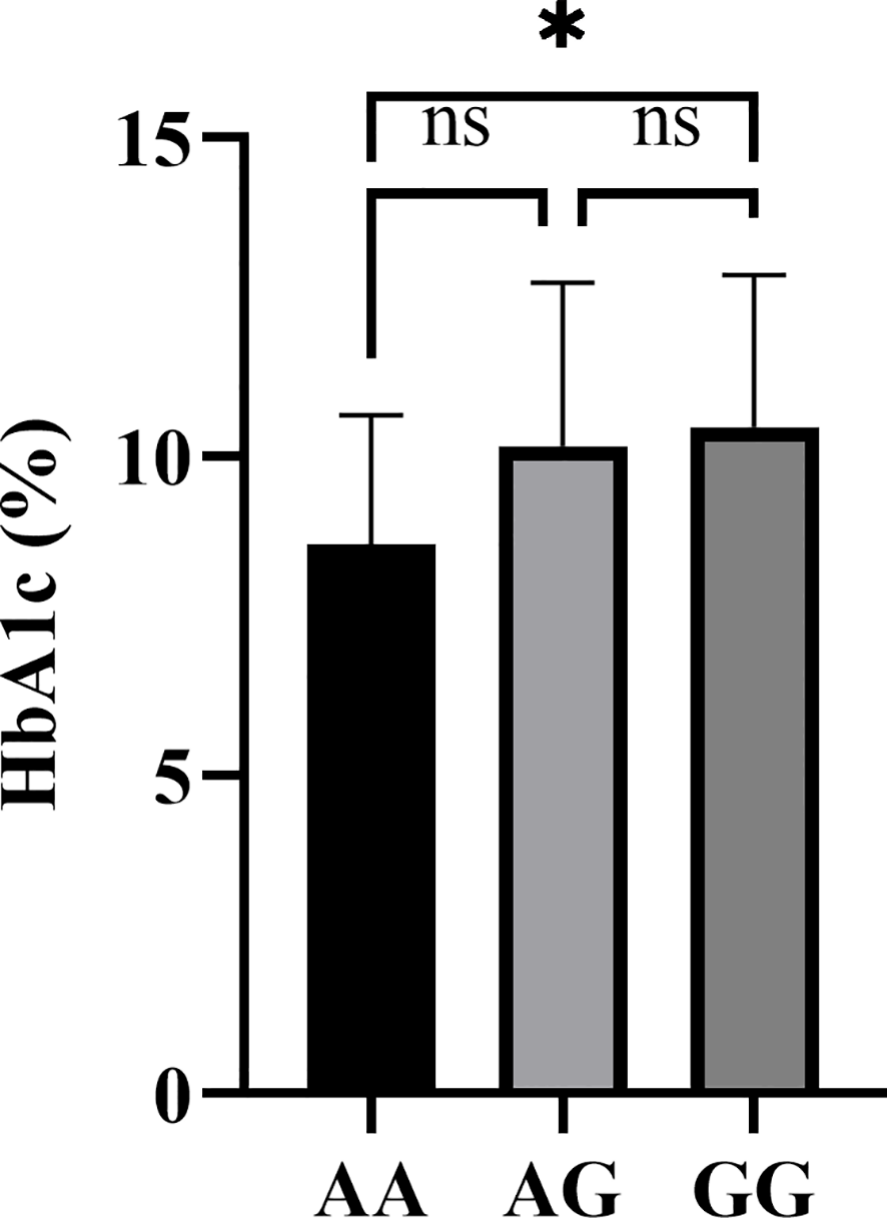
Multiple comparisons of HbA1c levels by rs2227982 genotypes in T1DM. The vertical axis represents HbA1c levels (%). Data points indicate individual measurements for each genotype group (AA, AG, GG). Statistical significance between genotypes (e.g., AA vs. GG) is denoted by asterisks (*), with *P* < 0.05. Significance testing was performed using ANOVA followed by Bonferroni-adjusted *post hoc* comparisons.

In contrast, exploratory analysis of rs2227982 genotypes demonstrated a significant difference in HbA1c levels (*P* = 0.036) among AA (n=17), GA (n=38), and GG (n=34) genotypes (**Table 6**). Bonferroni-adjusted *post hoc* comparisons indicated that GG genotype carriers exhibited significantly higher HbA1c levels compared to AA genotype carriers (*P* = 0.036<0.05; **Figure 1**). Although FPG levels tended to be elevated in GG genotype patients versus AA carriers, this trend did not reach statistical significance (*P* = 0.060; **Table 6**). No significant correlations were observed between rs2227982 genotypes and autoantibody positivity rates (*P* = 0.203; **Table 6**). These findings suggest that the rs2227982 G allele may contribute to poorer glycemic control in T1DM patients.

**Table 6:** Clinical characteristics of T1DM patients with different genotypes at rs2227982.

Parameter	AA(n=17)	GA(n=38)	GG(n=34)	F	*P* value
HbA1c (%)	8.62 ± 2.02^1^	10.15 ± 2.57	10.46 ± 2.39^1^	3.467	0.036
FPG (mmol/L)	8.42 ± 5.34	11.82 ± 6.73	12.91 ± 6.3	2.902	0.060
Autoantibody positivity (n)	10 (58.8)	28 (73.7)	28 (82.4)		0.203

T1DM, type 1 diabetes mellitus; HbA1c, glycated hemoglobin; FPG, fasting plasma glucose. Continuous variables are presented as mean ± standard deviation (SD) and were compared using one-way analysis of variance (ANOVA). When the overall ANOVA was statistically significant (P < 0.05), Bonferroni-adjusted *post hoc* pairwise comparisons were performed. Categorical variables are presented as number (percentage) and were compared using the χ^2^ test or Fisher’s exact test where appropriate. Autoantibody positivity was defined as positivity for at least one of insulin autoantibody (IAA), islet antigen 2 autoantibody (IA-2A), and glutamic acid decarboxylase autoantibody (GADA). ^1^*P* < 0.05 for AA vs. GG comparison (Bonferroni *post hoc* test).

### 3.6 Comparative analysis of serum sPD-1 concentrations and clinical characteristics between T1DM patients and healthy controls

**Figure 2:**
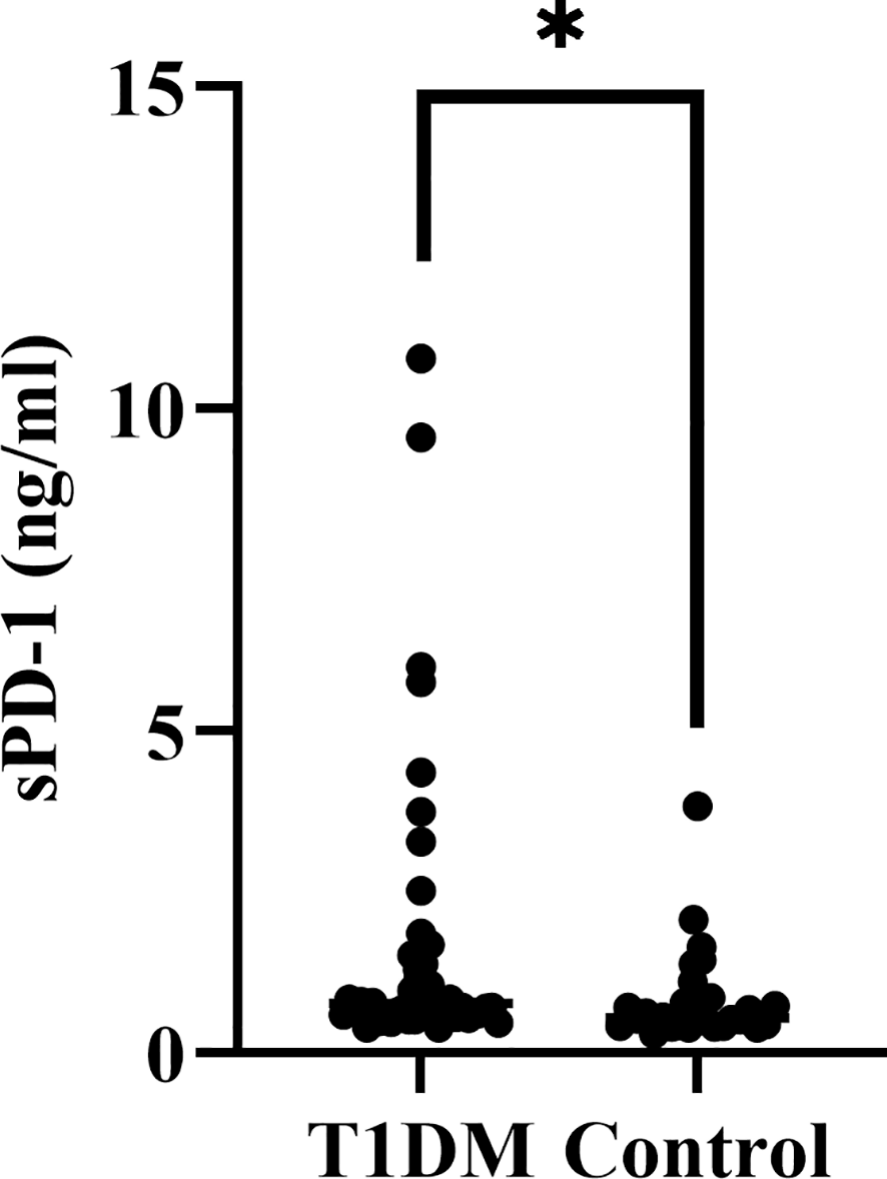
sPD-1 levels in T1DM and healthy controls. The vertical axis represents sPD-1 concentrations (ng/mL). The data points show individual measurements, with a significant increase observed in the T1DM group compared to controls, indicated by the asterisk (*). Statistical significance was determined using appropriate tests, showing *P* < 0.05.

Serum sPD-1 levels and metabolic parameters were evaluated in 44 T1DM patients and 28 age- and sex-matched healthy controls. T1DM patients exhibited significantly elevated sPD-1 concentrations compared to controls (*P* = 0.013) (**Figure 2**; **Table 7**). As expected, glycemic markers were markedly higher in T1DM patients, including HbA1c (*P* < 0.001) and FPG (*P* < 0.001). No significant intergroup differences were observed in age, sex, UA, ALT, AST, Scr, TG, TC, HDL-C, LDL-C (all *P >*0.05; **Table 7**).

**Table 7:** Concentration and clinical characteristics of serum sPD-1 in T1DM and healthy controls.

Parameter	T1DM group (n=44)	Control group (n=28)	*P* value
Age (years)	44.52 ± 18.65	43.46 ± 11.76	0.790
sPD-1(ng/ml)†	0.77 (0.61, 1.41)	0.55 (0.43, 0.80)	0.013
HbA1c (%)	9.92 ± 2.60	5.36 ± 0.26	<0.001
FPG (mmol/L)	11.12 ± 6.55	4.89 ± 0.36	<0.001
UA (μmol/L)	302.09 ± 91.31	309.11 ± 67.71	0.727
ALT (U/L)	16.36 ± 11.79	18.07 ± 5.23	0.473
AST (U/L)	17.86 ± 8.28	20.39 ± 4.26	0.140
Scr (μmol/L)	66.54 ± 56.87	64.66 ± 13.45	0.865
TG (mmol/L)†	1.12 (0.84, 1.65)	1.02 (0.92, 1.42)	0.361
TC (mmol/L)†	4.93 (3.90, 5.40)	4.46 (4.19, 4.76)	0.054
HDL-C (mmol/L)†	1.28 (1.13, 1.43)	1.23 (1.13, 1.48)	0.759
LDL-C (mmol/L)†	2.62 (2.01, 2.98)	2.87 (2.61, 2.97)	0.085
Sex, n(%)			0.786
male	20 (45.5)	11 (39.3)	
female	24 (54.5)	17 (60.7)	

sPD-1, soluble PD-1; T1DM, type 1 diabetes mellitus; HbA1c, glycated hemoglobin; FPG, fasting plasma glucose; UA, uric acid; ALT, alanine aminotransferase; AST, aspartate transferase; Scr, serum creatinine; TG, triglycerides; TC, total cholesterol; HDL-C, high-density lipoprotein cholesterol; LDL-C, low-density lipoprotein cholesterol. Notes: Data are presented as mean ± standard deviation (SD) for normally distributed variables, and between-group comparisons were performed using the independent-samples t test. Non-normally distributed variables (marked with †) are presented as median (interquartile range, IQR), and were compared using the Mann–Whitney U test. Categorical variables are expressed as number (percentage) and were compared using the χ^2^ test or Fisher’s exact test where appropriate.

### 3.7 Correlation analysis between serum sPD-1 levels and clinical parameters in patients with T1DM

In T1DM patients, serum sPD-1 levels were inversely correlated with age (r=−0.426, *P* = 0.004) and positively associated with UA (r=0.592, *P* < 0.001). In contrast, no correlations were observed between sPD-1 levels and sex, HbA1c, FPG, fasting C-peptide (FCP), Scr, ALT, AST, TG, TC, HDL-C, LDL-C, body mass index (BMI), GADA, or IAA (all *P >*0.05; **Table 8**).

**Table 8:** Correlation analysis between serum sPD-1 and clinical characteristics in T1DM.

Parameter	r	*P* value
Age (years)	-0.426	0.004
Sex (%)	0.253	0.097
HbA1c (%)	0.135	0.383
FPG (mmol/L)	-0.035	0.823
FCP (pmol/L)	-0.153	0.521
Scr (μmol/L)	-0.092	0.552
UA (μmol/L)	0.592	<0.001
ALT (U/L)	-0.145	0.347
AST (U/L)	-0.227	0.138
TG (mmol/L)	0.023	0.881
TC (mmol/L)	-0.066	0.668
HDL-C (mmol/L)	0.016	0.921
LDL-C (mmol/L)	0.056	0.737
BMI (kg/m2)	-0.310	0.843
GADA (IU/ml)	0.109	0.503
IAA (IU/ml)	-0.082	0.602

sPD-1, soluble PD-1; T1DM, type 1 diabetes mellitus; HbA1c, glycated hemoglobin; FPG, fasting plasma glucose; FCP, fasting C-peptide; Scr, serum creatinine; UA, uric acid; ALT, alanine aminotransferase; AST, aspartate transferase; TG, triglycerides; TC, total cholesterol; HDL-C, high-density lipoprotein cholesterol; LDL-C, low-density lipoprotein cholesterol; BMI, body mass index; GADA, glutamic acid decarboxylase autoantibody; IAA, insulin autoantibody. Notes: Pearson’s correlation was used for normally distributed continuous variables, while Spearman’s rank correlation was applied for non-normally distributed or ordinal variables. Correlation coefficients are presented as r.

**Figure 3:**
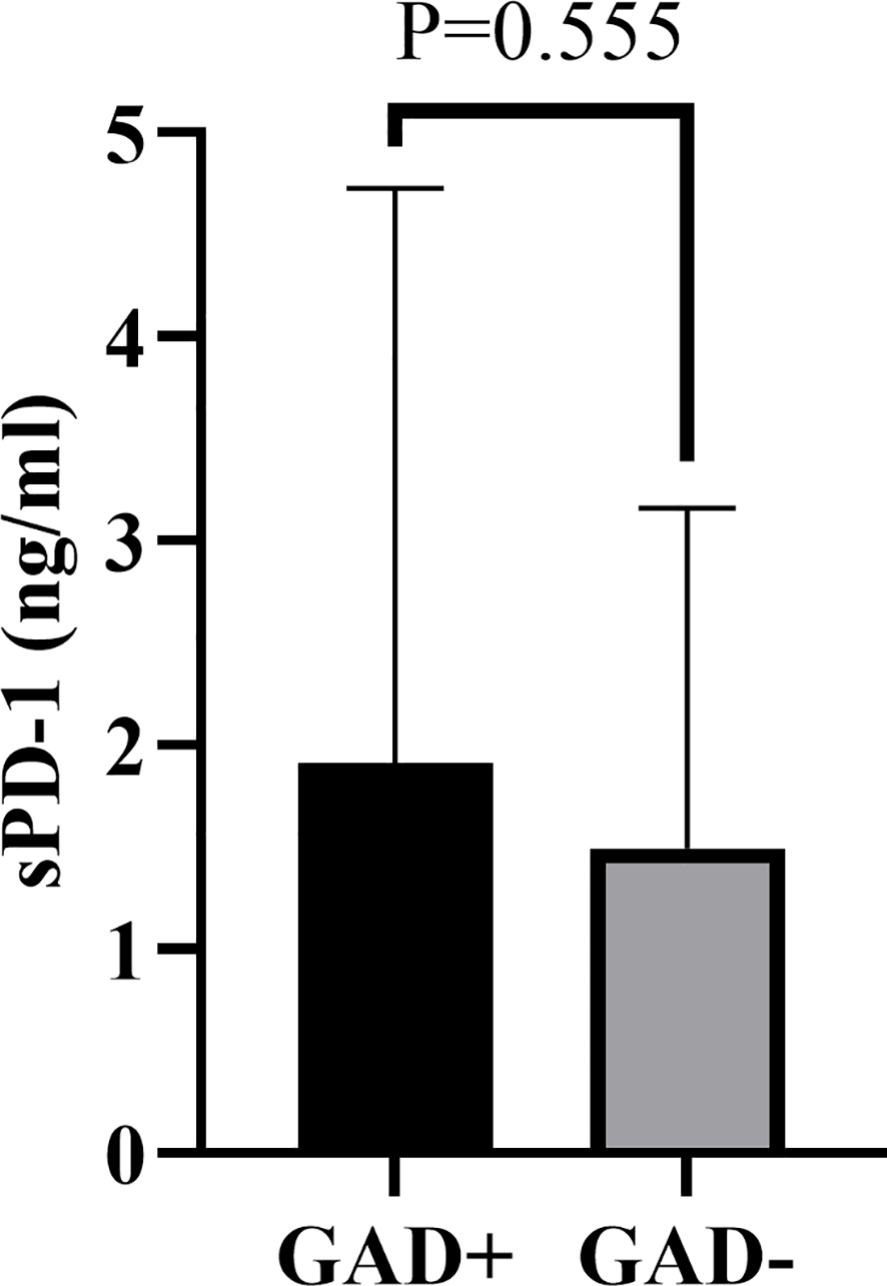
Comparison of serum sPD-1 levels in T1DM patients stratified by GADA positivity. The vertical axis represents sPD-1 concentrations (ng/mL). Individual data points are shown for GADA-positive and GADA-negative groups. Horizontal bars indicate group means. No statistically significant difference was observed between groups (*P* = 0.555). GADA=GAD, glutamic acid decarboxylase autoantibody.

**Figure 4:**
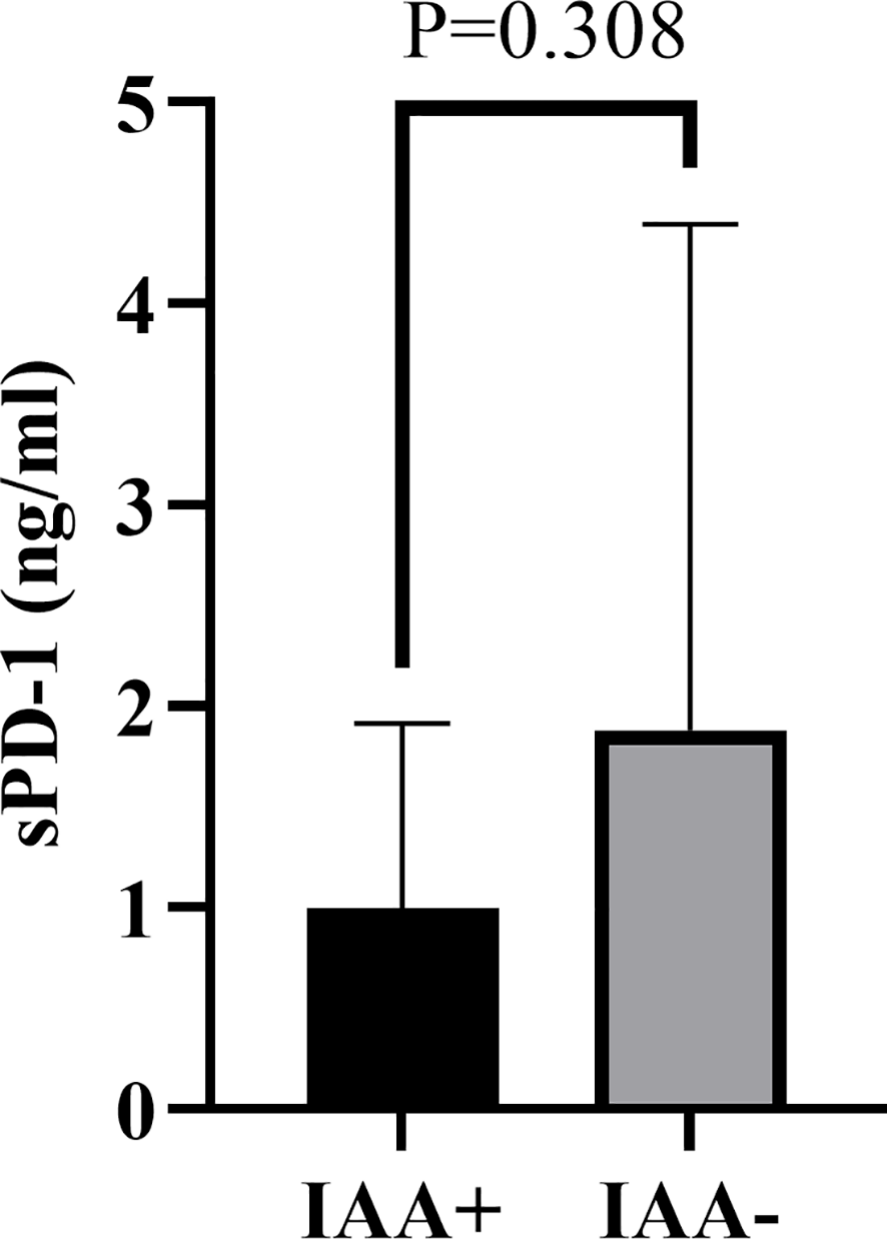
Comparison of serum sPD-1 levels in T1DM patients stratified by IAA positivity. The vertical axis represents sPD-1 concentrations (ng/mL). Individual data points are shown for IAA-positive and IAA-negative groups. Horizontal bars indicate group means. No statistically significant difference was observed between groups (*P* = 0.308). IAA, insulin autoantibody.

To further investigate the association between diabetes autoantibodies and sPD-1 levels in T1DM patients, participants were stratified based on GADA positivity. Comparative analysis revealed no statistically significant difference in sPD-1 levels between GADA-positive (1.909 ± 2.817 ng/mL) and GADA-negative groups (1.494 ± 1.665 ng/mL) (*P >*0.05, **Figure 3**). Similarly, stratification by IAA positivity showed no significant difference in sPD-1 levels between IAA-positive (0.998 ± 0.918 ng/mL) and IAA-negative groups (1.882 ± 2.511 ng/mL) (*P >*0.05, **Figure 4**).

### 3.8 Correlation between PD-1 polymorphisms and serum sPD-1 levels

No significant genotype-associated variations in serum sPD-1 levels were observed for either PD-1 rs2227981 or rs2227982 (both *P* > 0.05; **Table 9**).

**Table 9:** Comparison of serum sPD-1 levels by rs2227981 and rs2227982 genotypes in T1DM patients.

SNP	Genotype	sPD-1 (ng/mL)	*P* value
rs2227981	AA	0.806 (0.743, 3.432)	0.208
	GA	0.705 (0.625, 3.819)	
	GG	0.730 (0.539, 1.139)	
rs2227982	AA	0.906 (0.659, 2.307)	0.384
	GA	0.649 (0.540, 1.133)	
	GG	0.705 (0.621, 2.210)	

sPD-1, soluble PD-1; T1DM, type 1 diabetes mellitus; SNP, single nucleotide polymorphism. Serum sPD-1 levels are presented as median (interquartile range, IQR). Comparisons among groups were performed using the Kruskal–Wallis H test. As no statistically significant differences were observed, *post hoc* analyses were not conducted.

## 4 Discussion

Gene polymorphisms have been widely implicated in T1DM susceptibility across diverse populations. Nielsen et al. first identified PD-1 rs11568821 as a risk locus (OR = 1.92) in a Danish cohort of 94 T1DM patients through analysis of 14 PD-1 SNPs (26). Subsequent studies in Japanese, Chilean, and Chinese populations further demonstrated associations between multiple PD-1 variants, including rs2227981 and rs2227982, and T1DM susceptibility (23, 24, 29). Together, these findings support the contribution of PD-1 pathway genetic variability to autoimmune diabetes risk across ethnic groups.

In the present study, genotyping analysis of a Han Chinese cohort from Lianyungang, Jiangsu Province identified rs2227981 and rs2227982 polymorphisms as significant associated with T1DM susceptibility, consistent with prior reports in East Asian populations (23, 24).

The rs2227981 polymorphism, located in exon 5 (+7785 C/T), is a synonymous variant that does not alter the amino acid sequence of PD-1 (30). Its association with T1DM may reflect linkage disequilibrium with other functional variants within the PD-1 locus (31). Although no correlation between rs2227981 and clinical indicators was observed in our cohort, Fujisawa et al. reported genotype-dependent differences in PD-1 expression, suggesting potential regulatory effects (32).

The rs2227982 polymorphism, located in exon 5 (+7625 G/A), is a nonsynonymous variant resulting in a valine-to-alanine substitution (31). While valine and alanine share similar biochemical properties, subtle structural alterations may influence PD-1 signaling efficiency. In our study, rs2227982 was significantly associated with T1DM susceptibility, and carriers of the G allele exhibited higher HbA1c levels. However, this association should be interpreted cautiously given the cross-sectional design and limited sample size. Rather than implying a direct effect on glycemic control, the rs2227982 variant may reflect genetic influences on immune-regulatory processes that indirectly interact with metabolic status. Further functional studies are required to clarify the biological consequences of this substitution in the context of T1DM pathogenesis.

A previous study demonstrated that the rs4143815 polymorphism in the PD-1 gene is significantly associated with autoantibody positivity in individuals at risk for T1DM (22). However, we did not observe significant associations between rs2227981 or rs2227982 polymorphisms and autoantibody positivity (IAA or GADA). These findings suggest that the contribution of PD-1 variants to T1DM susceptibility may not be mediated through classical humoral autoimmunity pathways. However, IA-2A was excluded due to low prevalence, and larger cohorts are needed to confirm these observations.

PD-1 exists in both membrane and soluble forms (13, 33, 34). sPD-1 has been reported to exhibit functional antagonism, thereby inhibiting the immune regulatory effects of the membrane-bound PD-1 and PD-L1 (35). Elevated circulating sPD-1 levels have been described in several autoimmune and inflammatory diseases, sometimes correlating with disease activity (15–17, 27, 36).

In our study, serum sPD-1 levels were significantly elevated in T1DM patients compared with controls, suggesting altered immune checkpoint regulation. However, in our cross-sectional analysis, sPD-1 levels were not significantly associated with glycemic parameters, islet function markers, or diabetes-related autoantibodies. Therefore, sPD-1 cannot be considered a marker of metabolic control or overt autoimmune activity in this dataset. Instead, it may reflect a broader dimension of immune checkpoint modulation that operates independently of short-term metabolic burden. The clinical relevance of circulating sPD-1 as a disease activity indicator warrants further investigation in longitudinal studies.

Recent studies indicate that soluble immune checkpoint receptors such as sPD-1 can be generated via alternative splicing and are biologically active molecules capable of modulating immune responses in autoimmune settings, reflecting dynamic regulatory states beyond membrane-bound signaling (4, 37). Furthermore, PD-1 gene expression is subject to multi-level regulation at transcriptional and post-transcriptional stages, suggesting that genetic variants such as rs2227981 and rs2227982 may affect checkpoint signaling efficiency by modifying expression dynamics (12). The PD-1 signaling pathway plays pivotal roles in maintaining immune homeostasis and regulating tolerance, with dysregulation implicated in autoimmunity, thus providing a plausible mechanistic link between PD-1 axis disruption and β-cell autoimmunity in T1DM (38). These findings provide a broader mechanistic context for our observations, although functional validation was beyond the scope of the present study.

In our study, serum sPD-1 levels were inversely correlated with advancing age and positively associated with UA concentrations, highlighting potential roles of immunosenescence and metabolic dysregulation in modulating the PD-1 pathway. These findings suggest that both aging and metabolic factors may contribute to the regulation of immune checkpoint molecules such as sPD-1 in T1DM. However, considering the limited number of new-onset T1DM cases in our study, this conclusion needs to be further explored with a larger sample size. Future studies should focus on validating these observations and explore the underlying mechanisms that link age and UA to immune checkpoint dysregulation in autoimmune diseases like T1DM.

Several limitations should be noted. First, lipid-lowering medication use and detailed dietary/lifestyle management were not systematically recorded; therefore, the observed lower LDL-C levels in the T1DM group may have been influenced by unmeasured treatment or behavioral factors and should be interpreted cautiously. Second, disease duration was not collected as part of the original data collection framework, which limits our ability to assess time-dependent effects on metabolic and immunological measures. Future studies with standardized capture of medication exposure and disease duration are warranted to validate these findings. Third, the cross-sectional design precludes causal inference. Larger, longitudinal studies with standardized clinical data collection are required.

This study is novel in that it simultaneously evaluates both genetic variants of PD-1 (rs2227981 and rs2227982) and circulating sPD-1 levels in a single cohort of T1DM patients. This integrated approach allows for a more comprehensive understanding of the relationship between genetic susceptibility and immune dysregulation in T1DM. Additionally, we replicated PD-1 susceptibility signals in a Han Chinese population, which is an important contribution to validating these findings across different ethnic groups. Another novel aspect of this study is the identification of a potential association between the rs2227982 polymorphism and the HbA1c phenotype, suggesting that PD-1 gene variants may not only affect immune regulation but also glycemic control in T1DM. Finally, our study proposes that immune checkpoint dysregulation, as indicated by elevated sPD-1 levels, may occur independent of glycemic burden, providing preliminary evidence for immune checkpoint dysregulation in T1DM. However, given the cross-sectional design and limited sample size, these findings should be interpreted as exploratory rather than definitive mechanistic conclusions.

## 5 Conclusions

Our findings demonstrate that PD-1 rs2227981 and rs2227982 variants are associated with T1DM susceptibility in a Chinese Han cohort. The rs2227982 polymorphism was also linked to higher HbA1c levels, although causal interpretation is limited by the study design. Elevated circulating sPD-1 levels in T1DM patients suggest altered immune checkpoint regulation, but the lack of association with glycemic and autoantibody parameters indicates that sPD-1 may not reflect short-term disease activity. These results are exploratory and require confirmation in larger, longitudinal, and mechanistic studies.

## Data Availability

The original contributions presented in the study are included in the article/**Supplementary Material**. Further inquiries can be directed to the corresponding authors.
