# Supplementary material for: Association of PD-1 gene polymorphisms and serum soluble PD-1 levels with type 1 diabetes mellitus susceptibility in a Chinese Han cohort: a case-control study
Source: Front Immunol. 2026 Mar 27;17:1783770. doi: 10.3389/fimmu.2026.1783770 (PMC13066176; doi:10.3389/fimmu.2026.1783770)
Supplement: Supplementary file 1 [file DataSheet1.pdf]

Supplementary Material

Supplementary Table 1. Multivariable Regression Analysis of Factors Influencing Serum sPD-1 Levels

	B (Unstandardized Coeff.)	SE	Beta (Standardized Coeff.)	*t*	*P*	95% CI for B
Constant	-1.043	1.173		-0.889	0.377	[-3.384, 1.299]
T1DM	0.521	0.681	0.135	0.766	0.446	[-0.838, 1.880]
HbA1c	0.143	0.114	0.229	1.253	0.215	[-0.085, 0.372]
FPG	-0.031	0.045	-0.097	-0.678	0.500	[-0.122, 0.060]
LDL-C	0.461	0.348	0.157	1.326	0.189	[-0.233, 1.156]

Abbreviations: sPD-1, soluble PD-1; SE, standard error; T1DM, type 1 diabetes mellitus; HbA1c, glycated hemoglobin; FPG, fasting plasma glucose; LDL-C, low-density lipoprotein cholesterol; CI, confidence interval.
